# Effects of Monochromatic Blue Light on Reducing the Adverse Impact of Induced Cyclic Chronic Heat Stress during the Thermal Manipulation of Broiler Embryos

**DOI:** 10.1155/2022/9898311

**Published:** 2022-06-14

**Authors:** Li Zeng, Qiong Liu, Tao Wang, Yefeng Yang, Ahmed Jado, Yasser Elhadidi, Wenfeng Lin, Jian Li, Jinming Pan

**Affiliations:** ^1^Department of Biosystems Engineering, Zhejiang University, Hangzhou 310058, China; ^2^Department of Agricultural Engineering, Mansoura University, Mansoura 35516, Egypt

## Abstract

**Objective:**

The aim was to detect effects of blue light on reducing the adverse effect of heat stress in thermal manipulation (TM) of broiler embryos by subjecting embryos to heat stress during incubation development.

**Methods:**

Eggs were assigned to four treatments in which the TM (thermal manipulation) was exposed to 40°C for 4 h daily during five successive days, if TM was operated. The treatments were (1) normal temperature with white lighting group (37°C+W), (2) normal temperature with blue lighting group (37°C+B), (3) thermal manipulation with white lighting group (40°C+W), and (4) thermal manipulation with blue lighting group (40°C+B).

**Results:**

Blue light significantly lowered MDA and corticosterone concentrations in the embryonic liver. Additionally, the damage of embryonic liver tissue caused by heat stress could be reduced by blue light. HSPs and HSFs gene expression of chicken liver were modulated by blue light significantly, whereas the effects were different, respectively. Moreover, blue light modulated liver antioxidant enzyme activity and their gene expression in embryonic liver significantly. However, blue light did not exert significant effects on body weight, late hatch rectal temperature and tibia length of hatched chicks.

**Conclusions:**

The results suggest that monochromatic blue light can reduce the content of MDA and corticosterone of broiler embryos in heat stress and increase the relative expression of SOD and CAT genes. Moreover, the monochromatic blue light may reduce the metabolic heat production of broilers during the embryonic stage, thus reducing the damage of broilers due to heat stress during the embryonic heat acclimation stage.

## 1. Introduction

For the global poultry production, climate change has gradually become a huge challenge [1]. The pecuniary losses resulting from high temperature in the poultry industry alone is up to $128 to $165 million per year. The annual pecuniary losses of the livestock of the United States is 16.9 to23.6 million dollars [2]. Finding measures to reduce the harm caused by heat stress is very important for some tropical regions of the world. Heat treatment of broiler chickens during incubation by exposing the embryo to heat stress has been shown to increase the heat resistance of broilers in the later stage [3, 4]. Broilers hatched after embryonic exposure to intermittent TM have lower calorific rates, lower weight of abdominal fat pads, larger muscle growth, and a thinner average fibre diameter under heat stress (32°C for 12 h/d) [5]. In addition, compared with the control, metabolic rate and calorific value of TM treated embryos were lower [6]. However, heat exposure reduces animal welfare and growth performance [7]. Yolk consumption and embryo growth can be lowered by embryo TM [8]. Additionally, heat stress triggers oxidative stress in the liver, increasing tissue damage [9]. Elevated blood corticosterone concentration indicates that heat exposure increases the stress response [4].

Severity of heat stress is decided by degree of destruction of the balance between cellular antioxidants and reactive oxygen species (ROS) [10–13]. Both catalase (CAT) and superoxide dismutase (SOD) belong to cellular antioxidant enzymes which have different responses with different affected tissues [10]. Additionally, heat stress will cause protein damage, which will lead to the aggregation of unfolded proteins [14, 15]. The expressions of heat shock proteins (HSPs) are increased by heat stress, which leads to protein stability and heat resistance [16]. HSP70 and HSP90 are two kinds among the HSPs. HSP70 and HSP90 work to protect affected cells, preventing the aggregation of unfolded proteins [17]. Heat shock transcription factors (HSFs) can modulate HSP expression by acting on specific DNA sequence. (heat shock element (HSE)) [18–20].

Different mitigation methods have been adopted to reduce the harm caused by heat stress, which include environmental strategies in shape of housing, ventilation, and natural or artificial shading, along with feed additives [1]. Another approach that can alleviate the heat stress deleterious effects is lighting management. Abdo et al. [9] described that susceptibility of broilers could be reduced by blue light. Reducing the stress response and improving immune response in broilers has been proved to be the effect of blue light [21]. In addition, blue light may alleviate stress response by modulating expression of interleukin-1*β* (IL-1*β*) and ratio of heterophils to lymphocytes (H/L) [22, 23]. The target of the present study was exploring function of blue light in alleviating adverse effect of thermal manipulation by exposing embryos to periodic high temperature during incubation development.

## 2. Materials and Methods

### 2.1. Incubation Management

Hatching eggs (*n* = 800) were selected by weight (60 ± 2 g) from Huangjiaoma broiler breeders. The breeders were from a company at Jiaxing, China. All eggs were incubated in separate environmentally controlled commercial incubators from a company at Shandong, China. The incubators were set at the incubation room, Zhejiang University, China. The relative humidity (RH) of the incubators was kept at 60 ± 1%, and temperature of the incubator was maintained at 37.8 ± 0.1°C. Eggs were turned from D1 to D18 with 2 h a day. Eggs were examined on 18th day to remove the nonviable eggs.

### 2.2. Experimental Design

Before incubation, the hatching eggs were disinfected and equally divided into four groups (*n* = 200), with four replicates in each group (*n* = 50). Two groups (W+37.6°C and B+37.6°C) were incubated at normal temperature under white light or blue light. The other two groups (W+40°C and B+40°C) received specific TM. On this point, TM was carried out from 14th day to 18th day in which eggs were exposed to 40 ± 1°C for 4 h a day. After TM, the temperature was lowered to 37.6°C till hatch. The intensity of blue light was 27 lux and the wavelength was 450 nm.

### 2.3. Sample Collection

Twenty-four eggs were randomly taken from each group for specimen gathered after TM (after day 18 heat acclimatization). Cervical dislocation was used to kill the embryos before liver specimens of each bird were collected. Formalin (10%) was used to preserve one-third of liver specimens for histopathological examination; for RNA extraction, one-third of liver specimens were frozen in liquid nitrogen; for antioxidant enzyme activity analysis, one-third of liver specimens were preserved at −20°C.

### 2.4. Histopathological Examination

The tissues were treated with paraffin. Each specimen was sectioned and mounted on a slide. The tissues were stained with hematoxylin and eosin (H&E), and then, the liver sections were examined with a light microscope (200x) [24].

### 2.5. Corticosterone and Malondialdehyde (MDA) Content Determination

Liver samples were placed in phosphate buffer (pH 7.2-7.4). The supernatant was obtained by centrifuging liver homogenate (5000 rpm at 4°C for 15 min). The concentration of malondialdehyde was measured using a biological diagnostic kit (Biodiagnostic, A003-1, China). The content of malondialdehyde was determined by ultraviolet visible spectrophotometer at 532 nm. Liver corticosterone content was assayed using corticosterone ELISA kit (Biodiagnostic, MB-5253A, China), measuring absorbance (450 nm) by spectrophotometer (Ahmed et al., 2020).

### 2.6. Antioxidant Enzyme Activity Determination

SOD activity was measured using diagnostic kit (Biodiagnostic, MB-9428A, China). CAT activity was determined by spectrophotometry based on the spectrophotometric using diagnostic kits (MB-9429A, China), measuring absorbance (450 nm).

### 2.7. RNA Extraction and cDNA Synthesis

RNA was extracted from the liver tissue (30 mg) (*n* = 8 from each treatment) by RNA purification kit (Norgen Biotek Corporation, Canada). RNA integrity was verified by gel electrophoresis of rRNA strips. Additionally, UV-Vis spectrophotometer (Nanodrop ND1000) was used to determine the concentration of RNA. RNA sample was reverse transcribed finally.

### 2.8. Real-Time PCR

Real-time PCR (qPCR) was performed using the QuantiFast SYBR® Green Supermix (Qiagen, Germany). The reaction mixture included 10 *μ*l of 2×SYBR Green, 2 *μ*l of cDNA, and 0.5 *μ*M of each prime. All the genes were denatured initially at 95°C for 15 min followed by 40 cycles at 95°C for 15 s and annealed at 60°C for 1 min. Dissociation curve analysis started at 65°C and ended at 95°C to verify the specificity of PCR products (with an increase of 0.5°C every 5 s). There was only one peak in the dissociation curve analysis of all tested genes at a specific melting temperature, indicating the specificality of the PCR product amplification. Genes of eight birds were tested repeatedly. The “fold change” calculation included CT values of each sample [25].

### 2.9. Statistical Analysis

The data were analyzed by GraphPad Prism 7 software. Statistically significant differences of temperature and light treatment effects on MDA, corticosterone, antioxidant enzymes and gene expression of *HSPs* and *HSFs*, and embryonic body temperature were detected by two-way ANOVA.

## 3. Results

### 3.1. Blue Light Significantly Lowers MDA and Corticosterone Concentrations in Embryonic Liver

**Figure 1:**
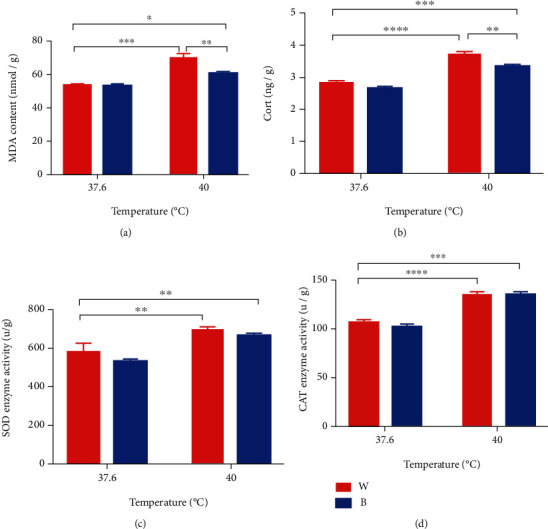
Concentration of MDA and corticosterone as well as activities of antioxidant enzymes in the liver of chick embryo under different conditions: (a) MDA content, (b) corticosterone concentration, (c) activity of SOD, and (d) activity of CAT. Mean ± SEM is shown. ∗, ∗∗, ∗∗∗, and ∗∗∗∗ denote statistical significance (two-way ANOVA) with *P* < 0.05, *P* < 0.01, *P* < 0.001, and *P* < 0.0001, respectively.

The effects of light and temperature on MDA concentration were statistically significant (*P* < 0.0001 for light and *P* < 0.05 for temperature). Because MDA is an index of lipid peroxidation, MDA level (Figure 1(a)) increased significantly in W+40°C and B+40°C compared to W+37.6°C in chick embryos (for W+40°C *P* < 0.001 and for B+40°C *P* < 0.05). Significantly, compared to W+40°C, the concentration of MDA was lower in B+40°C (*P* < 0.01).

The effects of light on corticosterone contents were significant (*P* < 0.0001 for light and *P* = 0.001 for temperature). The content of corticosterone (Figure 1(b)) increased significantly in W+40°C and B+40°C compared to W+37.6°C (for W+40°C *P* < 0.0001 and for B+40°C *P* = 0.0001). Significantly, compared with W+40°C, B+40°C induced a lower concentration of corticosterone (*P* < 0.01).

### 3.2. Blue Light Can Alleviate the Increase of Liver Interstitial Cracks Caused by Heat Stress

**Figure 2:**
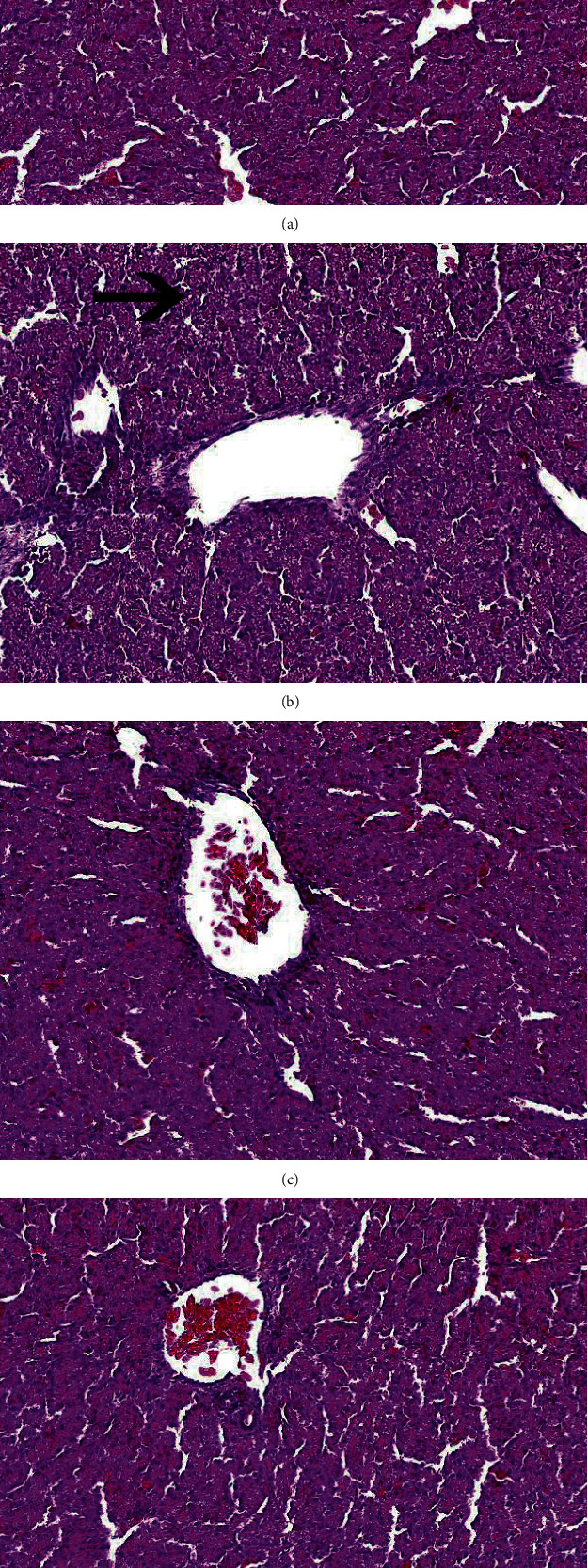
Liver histomorphology of Huangjiaoma chicken: (a) white light (W+37.6°C), (b) white light+H (W+40°C), (c) blue light (B+37.6°C), and (d) blue light+H (B+40°C). Arrows point the interstitial cracks.

Figures 2(a) and 2(c) show that the cells of embryonic liver were closely arranged and structurally intact in the case of W+37.6°C and B+37.6°C.  Figure 2(b) shows that there were more cracks and loose arrangement in embryonic liver tissue in the case of W+40°C; moreover, severe mononuclear cell infiltration and focal necrosis were found, which was consistent with the discovery of Abdo et al. [9] that heat stress under high temperature can lead to some degree of fat changes, perivascular monocyte infiltration, and focal necrosis.  Figure 2(d) shows that there were fewer cracks and the liver tissue structure was more compact in the case of B+40°C compared to W+40°C.

### 3.3. Gene Expression of *HSPs* and *HSFs* Can Be Modulated by Blue Light Significantly in Chicken Liver

**Figure 3:**
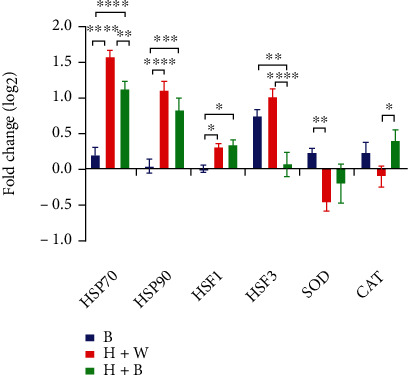
Expression of *HSPs*, *HSFs*, and antioxidant enzyme genes. The expression levels were presented as log2 fold change and shown in the figure as mean ± SEM. ∗, ∗∗, ∗∗∗, and ∗∗∗∗ denote statistical significance (two-way ANOVA) with *P* < 0.05, *P* < 0.01, *P* < 0.001, and *P* < 0.0001, respectively.

The damage of heat stress to liver could be reduced by blue light by reducing the concentration of MDA and corticosterone in chicken embryo (Figures 1(a) and 1(b), resp.).  Figure 3 presents the relative gene expression (compared to W+37.6°C) profiles of *HSPs* and *HSFs* from 8 birds for each treatment. The expression levels of *HSP70* and *HSP90* were regulated by blue light in chick embryos without temperature differences (*P* < 0.0001).

In the liver, heat stress significantly increased *HSP70* gene expression in W+40°C and B+40°C, whereas blue light at normal temperature only slightly increased its expression (for W+40°C *P* < 0.0001 and *P* < 0.0001 for B+40°C). Interestingly, in B+40°C, compared with W+40°C, the expression of *HSP70* gene was significantly downregulated by using blue light (*P* = 0.0094).

The expression pattern of *HSP90* (Figure 3) is similar to that of *HSP70*. There was no temperature difference, only the difference caused by light treatment, and there was no interaction between light and temperature (*P* > 0.05 for interaction and *P* < 0.0001 for light treatment). B+ 37°C upregulated gene expression level of *HSP90* insignificantly. In addition, compared with normal condition, W+40°C and B+40°C stimulated more *HSP90* gene expression (*P* < 0.0001 and *P* = 0.0003, resp.). However, in comparison with W+40°C, the expression of *HSP90* gene in B+40°C did not decrease significantly (*P* > 0.05).

For *HSF1* gene, difference due to temperature+light interaction was not significant (*P* > 0.05). W+40°C upregulated the expression of *HSF1* significantly, but the change was not significantly different with that induced by B+40°C (*P* = 0.7444). Furthermore, B+37.6°C nonsignificantly upregulated the expression levels of *HSF1* (*P* = 0.9534).

For *HSF3* gene, interaction of two conditions had a significant effect (*P* < 0.0001). W+40°C increased expression of *HSF3* significantly, whereas a slight increase appeared in B+40°C. Nevertheless, in B+37.6°C, it induced a significant increase (*P* = 0.0007).

To sum up, the expression of *HSPs* and *HSFs* can be regulated by blue light under heat stress. Changes that occurred on *HSP70* and *HSP90* were similar. However, changes of gene expression of *HSF1* and *HSF3* have different patterns.

### 3.4. Blue Light Modulates Activities and Gene Expression of Antioxidant Enzymes Significantly in Embryonic Liver

Figures 1(c) and 1(d), respectively, show the effects of different temperature and light conditions on antioxidant enzyme activity. Compared with W+37.6°C, the activity of SOD increased significantly in W+40°C and B+40°C (for W+40°C *P* < 0.01 and for B+40°C *P* < 0.01). However, in B+37.6°C, it caused a slight downregulation (*P* = 0.1168). In addition, CAT enzyme also had similar response after heat stress. CAT activity increased significantly in W+40°C and B+40°C compared to W+37.6°C (for W+40°C *P* = 0.0007 and for B+40°C *P* = 0.0004).

The expression levels of mRNA of antioxidant enzymes were measured to determine their regulatory patterns. For *SOD* (Figure 3), light treatment had significant difference, while for CAT, temperature effect had significant difference (with regard to SOD, temperature, and interaction treatment *P* > 0.05, light treatment *P* = 0.0112; with regard to CAT, temperature treatment *P* = 0.0214; light and interaction treatment *P* > 0.05). SOD gene expression was upregulated in B+37.6°C. Compared with W+40°C, the upregulation was significant, while heat stress stimulated a nonsignificant downregulation (*P* = 0.0543).

The reaction of CAT gene and SOD gene is different. Blue light caused a slight upregulation in B+37.6°C and significant upregulation in B+40°C (*P* = 0.1298 and *P* < 0.0239, resp.) compared to W+40°C. To sum up, blue light regulated the gene expression and the activity of antioxidant enzymes in the period of heat stress.

### 3.5. Blue Light Changed the Metabolic Performance and Development of Chick Embryo after Heat Stress and before Hatching

**Figure 4:**
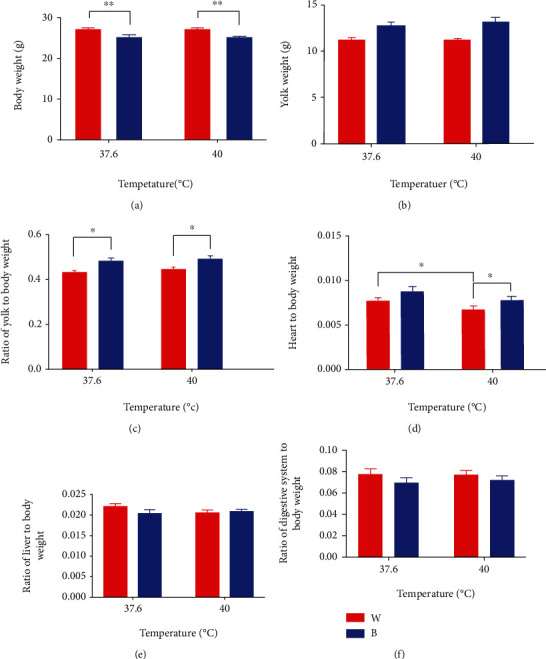
Metabolic performance and development of chick embryo following thermal manipulation (mean ± SEM): (a) body weight, (b) yolk weight, (c) weight ratio (yolk to body), (d) weight ratio (heart to body), (e) weight ratio (liver to body), and (f) weight ratio (digestive system to body). ∗ and ∗∗ denote statistical significance (two-way ANOVA) with *P* < 0.05 and *P* < 0.01, respectively.

Figure 4 shows multiple metabolic parameters and developmental status of chick embryo following thermal manipulation.  Figure 4(a) shows that body weight displayed a significant decrease in B+37.6°C compared to W+37.6°C (*P* < 0.01). Similarly, body weight displayed a significant decrease in B+40°C compared to W+40°C (*P* < 0.01). According to Figure 4(c), a significant increase of the ratio of yolk to body weight was noticed in B+37.6°C compared to W+37.6°C (*P* < 0.05). Similarly, the weight ratio (yolk to body) displayed a significant increase in B+40°C compared to W+40°C (*P* < 0.05). Besides, Figure 4(d) shows that heart to body weight displayed a significant increase in W+37.6°C and B+40°C compared to W+40°C (*P* < 0.05). Besides, no differences were observed between treatments in yolk weight and weight ratio (liver to body or digestive system to body) (*P* > 0.05).

### 3.6. Blue Light Did Not Affect Body Weight, Rectal Temperature, and Tibia Length of Hatched Chicks

**Figure 5:**
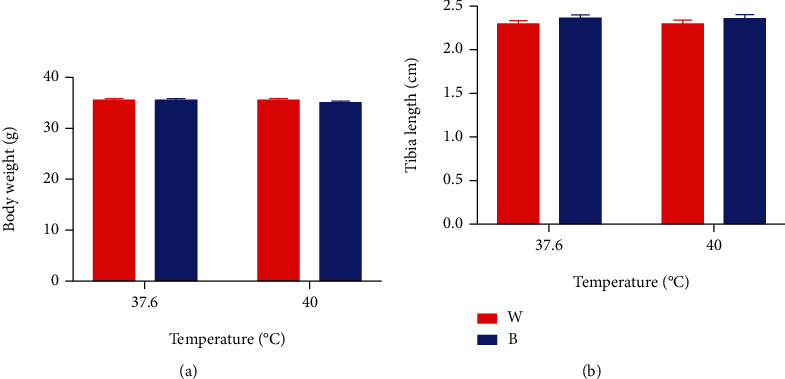
Body weight and tibia length of chicks after hatching (mean ± SEM): (a) body weight and (b) tibia length.

**Figure 6:**
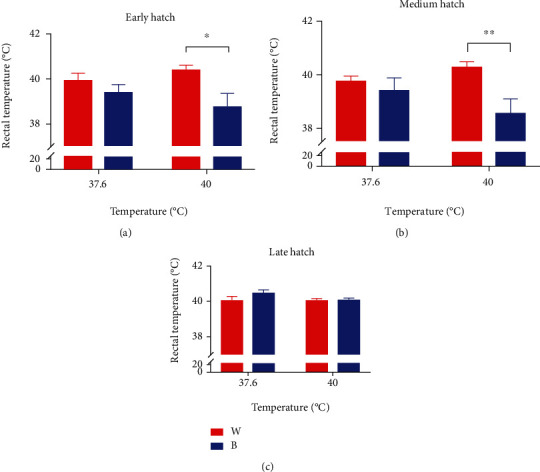
Rectal temperature of chicks after hatching (mean ± SEM): (a) early hatch, (b) medium hatch, and (c) late hatch. ∗ and ∗∗ denote statistical significance (two-way ANOVA) with *P* < 0.05 and *P* < 0.01, respectively.

The body weight of broilers is measured just after hatching. The body weight is presented in Figure 5(a). Differences (*P* > 0.05) between treatments in body weight were not observed. Figures 6 and 5(b) show the rectal temperature and tibia length of posthatch 0-day chicks. No differences (*P* > 0.05) in the date of late hatch rectal temperature and tibia length were observed between treatments.

## 4. Discussion

Meat type chickens have limited ability to face the destructive effect of heat stress. Several studies addressed heat challenge during embryogenesis enhancing adaptive capacity of broilers to cope with heat exposure. However, the heat challenge during embryonic development may trigger oxidative stress and stress response and reduce growth performance [8]. This research was aimed at exploring the effect of blue light on reducing the negative effect of TM during incubation.

With regard to MDA (Figure 1(a)), the content was significantly reduced in B+40°C compared to W+40°C. High MDA content is an indicator of lipid peroxidation, which can cause greater oxidative damage [26]. Under the condition of B+40°C, the concentration of MDA decreased, indicating that blue light may play a role in reducing negative effect caused by heat stress [27]. In addition, corticosterone (Figure 1(b) had similar response. Neuroendocrine system activities of poultry could be altered by heat stress, which contribute to activation of the hypothalamic–pituitary–adrenal axis. This disturbance causes elevation of corticosterone levels and degradation of the health status of animals [28, 29]. Elevated corticosterone levels of meat type chickens have demonstrated depressed lymphocyte number culminating in a higher H:L ratio [30]. The content of corticosterone is a sensitive indicator of stress in broilers and it is commonly used as a tool to assess the physiological stress [31]. The results of the present research indicated that exposure of eggs to heat stress significantly enhanced MDA and corticosterone content in embryonic liver, whereas supplementation of blue light in the environment was shown to restore the MDA and corticosterone level toward normal.

The effect of exposure to monochromatic blue light on liver histology was examined. Heat stress (W+40°C) caused tissue damage and increased interstitial fissures. Blue light (B+40°C) led to the loss of tissue damage compared with white light (W+40°C). Blue light can improve the resistance of broilers [23].

In the present research, when ambient temperature was normal, compared with W+37.6°C, B+37.6°C could significantly increase the expression of *HSP70* and *HSF3* genes, and these proteins acting as molecular chaperones to prevent protein aggregation can improve cell heat tolerance to resist high temperature, thus regulating the balance of cell survival and death. It is worth noting that compared with the condition of W+40°C, the gene expression of *HSP70*, *Hsp90*, and *HSF3* in B+40°C decreased slightly, which may be due to the fact that the way of improving the heat resistance of broilers by blue light is not single, and the need for heat shock protein of embryo was reduced due to improvement of heat stress resistance under the monochromatic blue light.

Figure 1 shows that activity of SOD and CAT was higher in high temperature group (40°C) than that in normal group (37.6°C), but the activity of the two enzymes was not changed by blue light significantly, which indicated that blue light may not help to regulate the activity of antioxidant enzymes. However, according to Figure 3, monochromatic blue light could increase the relative gene expression of two antioxidant enzymes. Especially, under high temperature, the gene expressions of the two enzymes were significantly decreased, but under this condition, compared with W+40°C, B+40°C also increased the gene expression of the two enzymes. This indicated that monochromatic blue light can improve the heat tolerance of broiler embryos through modulating gene expression of antioxidant enzymes rather than their activity.

According to Figure 4, a lower body weight of embryos under blue light could be found, compared to white light with corresponding temperature. In addition, at the same ambient temperature, blue light exposure meant a higher weight percentage of egg yolk than white light exposure, which indicated that monochromatic blue light might reduce the heat production of broiler embryos by reducing the metabolic rate of broiler embryos, because egg yolk was the main energy source of hatching embryos. However, after hatching, there was no difference in body weight between blue light group and white light group, and there was no difference in tibia length. In addition, according to Figure 6, the rectal temperature of broilers in B+40°C was significantly lower than that in W+40°C at the early and middle stage after hatching, and the rectal temperature directly represented the body temperature, which may also prove that blue light can reduce the heat production of hatching embryos. This difference did not exist at the late stage after hatching.

## 5. Conclusions

The results suggest that monochromatic blue light can reduce the content of MDA and corticosterone in broiler embryos after TM and increase the relative expression of SOD and CAT genes. Moreover, the monochromatic blue light may reduce the metabolic heat production of broilers during the embryonic stage, thus reducing the damage of broilers due to heat stress during the embryonic heat acclimation stage.

## Data Availability

The data that support the findings of this study are openly available in Figshare repository (10.6084/m9.figshare.17000221.v1).
